# Targeting aging hallmarks in brain health within the framework of preventive medicine: mechanistic insights into naringenin’s role in longevity, synaptic function, and cellular homeostasis

**DOI:** 10.3389/fnut.2025.1680349

**Published:** 2026-01-08

**Authors:** Yong Zhi, Shanshan Xie, Yanting Sun

**Affiliations:** 1 TCM Department, Xinjiang 474 Hospital, Urumqi, Xinjiang, China; 2 Xinjiang Key Laboratory of Mental Development and Learning Science, Xinjiang Normal University, Urumqi, Xinjiang, China; 3 Independent Researcher, Nanjing, China

**Keywords:** aging, brain health, naringenin, synaptic function, cellular homeostasis

## Abstract

Aging is the primary driver of neurodegenerative vulnerability and functional decline in the brain. This narrative review synthesizes preclinical *in vitro* and *in vivo* evidence on naringenin, a citrus-derived flavonoid with pleiotropic effects on aging-related pathways. Naringenin modulates oxidative stress, enhances mitochondrial efficiency, and supports neurotrophic signaling linked to synaptic integrity and cognitive resilience. Experimental studies consistently show activation of Nrf2, SIRT1, and PI3K/Akt pathways, attenuation of neuroinflammatory cascades, and restoration of redox and proteostatic balance. Through these integrated actions, naringenin targets several hallmarks of brain aging, including mitochondrial dysfunction, oxidative stress, and impaired cellular homeostasis. From a preventive medicine perspective, understanding these mechanisms may guide the development of safe, dietary interventions aimed at promoting healthy brain aging and delaying neurodegenerative processes.

## 1 Introduction

Aging is a gradual and inevitable biological process that affects all living organisms. It is characterized by a progressive decline in physiological function and adaptive capacity, increasing vulnerability to diseases and ultimately leading to death. Although aging is universal and intrinsic to life itself, the biological mechanisms underlying this process remain incompletely understood. It is now well established that aging manifests at multiple levels, from molecular and cellular to systemic, and its effects are influenced by genetic factors, environmental exposures, developmental history, and disease burden (1–3). The impact of aging is particularly evident in organs that are highly energy-dependent, such as the brain and heart. In the nervous system, aging contributes to cognitive decline and is the principal risk factor for neurodegenerative disorders like Alzheimer’s disease (AD) and Parkinson’s disease (PD). Similarly, the cardiovascular system experiences structural and functional deterioration with age, leading to increased incidence of heart failure, arrhythmias, and ischemic damage (4–6). As global life expectancy rises, age-related disorders have emerged as a critical public health concern, imposing substantial personal and societal costs (7). Multiple theories have been proposed to explain aging, including evolutionary hypotheses such as antagonistic pleiotropy and the disposable soma theory, which suggest trade-offs between reproductive success and long-term maintenance (8, 9). At the mechanistic level, several interconnected hallmarks have been identified as central to the aging process.

These include genomic instability, mitochondrial dysfunction, telomere attrition, epigenetic drift, impaired proteostasis, deregulated nutrient sensing, cellular senescence, stem cell exhaustion, and altered intercellular communication. Together, these hallmarks provide a framework for understanding the biological deterioration associated with age and for identifying targets for intervention (10, 11). One of the most consistent features of aging at the cellular level is the accumulation of oxidative stress. Reactive oxygen species (ROS), which are generated primarily as by-products of mitochondrial respiration, can damage lipids, proteins, and DNA. Although cells possess a complex antioxidant defense system, including enzymes like superoxide dismutase (SODs), catalase (CAT), and glutathione peroxidase (GPx), persistent oxidative stress overwhelms these systems and contributes to cellular senescence and organ dysfunction. In the brain, increased ROS and reactive nitrogen species are strongly linked to the decline in cognitive and motor function commonly observed in elderly individuals, even in the absence of overt disease (12, 13). Mitochondria are both a source and a target of oxidative stress, and their dysfunction plays a pivotal role in aging and related diseases. Age-associated mitochondrial alterations include reduced oxidative phosphorylation efficiency, accumulation of mitochondrial DNA mutations, and impaired dynamics of fusion and fission. These dysfunctions lead to decreased energy production, increased ROS generation, and activation of pro-apoptotic pathways (14, 15). In neurodegenerative diseases such as AD and PD, mitochondrial impairment contributes to hallmark pathologies, including amyloid-beta (Aβ) accumulation, tau hyperphosphorylation, and dopaminergic neuronal loss. Similar mitochondrial defects in cardiac tissue contribute to myocardial aging and increased susceptibility to stress and ischemia (16).

The deterioration of protein quality control systems with age also contributes to the aging phenotype. Proteostasis, the maintenance of cellular protein homeostasis, is maintained by two major systems: The ubiquitin-proteasome pathway and the autophagy-lysosome pathway (17). Aging is associated with impaired proteasomal activity and diminished autophagic flux, leading to accumulation of damaged or misfolded proteins. This, in turn, amplifies cellular dysfunction and contributes to the pathology of age-related diseases (18). For instance, in the brain, impaired autophagy has been implicated in the aggregation of toxic proteins in AD and PD (19), while in peripheral tissues, disrupted proteostasis contributes to tissue degeneration and inflammation (20). Another prominent feature of aging is chronic low-grade inflammation, often referred to as “inflammaging.” This pro-inflammatory state is driven by the accumulation of senescent cells that adopt a senescence-associated secretory phenotype (SASP), secreting cytokines, chemokines, and proteases that disrupt tissue architecture and function (21). In the brain, increased activity of microglia and astrocytes with age leads to exaggerated inflammatory responses to peripheral immune signals. This neuroinflammatory environment exacerbates neuronal vulnerability and accelerates the progression of neurodegenerative diseases. Moreover, aging alters communication between the brain and immune system, increasing the risk of mental health complications following immune activation (22, 23).

Despite extensive research on oxidative stress and mitochondrial dysfunction in aging, relatively few reviews have integrated these processes with neurotrophic regulation and synaptic maintenance. A clearer understanding of how redox imbalance influences neurotrophin signaling, particularly brain-derived neurotrophic factor (BDNF) and nerve growth factor (NGF), is essential, as these molecules orchestrate neuronal survival, synaptic remodeling, and cognitive resilience. Declining BDNF and NGF levels are strongly associated with age-related cognitive impairment and neurodegenerative risk, linking oxidative stress to structural and functional deterioration of the brain (24, 25). This review therefore aims to synthesize current evidence on naringenin’s role in modulating aging-related mechanisms, emphasizing its impact on oxidative, mitochondrial, and neurotrophic pathways as interconnected determinants of brain health.

Indeed, given the multifactorial nature of aging and its contribution to a broad range of chronic conditions, there is growing interest in identifying safe, dietary compounds that may mitigate the biological impacts of aging. Polyphenols, particularly those derived from fruits and vegetables, have attracted attention due to their antioxidant, anti-inflammatory, and neuroprotective properties (26). Among these, naringenin (NAR), a flavanone predominantly found in citrus fruits, has emerged as a promising candidate for targeting age-related dysfunction. NAR and its glycoside precursor, naringin, exhibit diverse biological effects, including modulation of redox status, attenuation of inflammatory responses, and support of mitochondrial function.

Experimental studies have demonstrated that NAR enhances the activity of antioxidant enzymes such as heme oxygenase-1 (HO-1) and NAD(P)H quinone dehydrogenase 1 (NQO1), while reducing markers of oxidative damage. NAR also activates signaling pathways such as PI3K/Akt and Nrf2/ARE, which are known to regulate cellular defense responses and promote survival under stress conditions (27). In the context of neurodegeneration, NAR has shown the ability to reduce Aβ deposition, decrease microglial activation, and inhibit pro-inflammatory cytokines in models of AD. It has also been observed to prevent dopaminergic neuron degeneration in PD models by modulating mitochondrial dynamics and preserving mitochondrial membrane potential. These effects appear to be mediated, at least in part, by the activation of the sirtuin 1 (SIRT1) enzyme, a NAD+-dependent deacetylase implicated in aging, energy metabolism, and cellular resilience (28, 29). Beyond the brain, NAR exerts protective effects in other tissues commonly affected by aging. In the cardiovascular system, it has been shown to enhance mitochondrial bioenergetics, reduce fibrotic remodeling, and improve cardiac performance in aged animal models. Its ability to activate mitochondrial potassium channels (mitoBK) and preserve myocardial function under ischemic conditions highlights its potential as a cardioprotective agent in elderly populations (30). Pharmacokinetic studies reveal that NAR and its metabolites are widely distributed in aged organisms, accumulating in the liver, kidneys, lungs, and central nervous system. Age-related changes in absorption, metabolism, and tissue distribution must be considered when assessing the therapeutic potential of NAR in older populations (31).

Nonetheless, its natural origin, safety profile, and broad spectrum of activity make it an attractive candidate for further investigation in the context of healthy aging and age-associated disease prevention. In summary, aging is a complex and multifaceted process that underlies the pathogenesis of many chronic diseases. Mitochondrial dysfunction, oxidative stress, neuroinflammation, cellular senescence, and proteostasis disruption are key contributors to this decline (32). NAR a citrus-derived polyphenol, demonstrates potent antioxidant, anti-inflammatory, and mitochondrial-supportive properties across various preclinical models. Its capacity to modulate central aging pathways positions it as a compelling nutraceutical candidate for promoting healthy aging and protecting against age-related neurodegeneration and systemic decline.

Therefore, this narrative review integrates mechanistic and preclinical evidence on naringenin’s role in modulating aging-related pathways, with particular focus on brain health, synaptic plasticity, and systemic homeostasis. Literature was identified through searches of PubMed, Scopus, and Web of Science up to September 2025 using combinations of the terms naringenin, naringin, aging, oxidative stress, mitochondria, autophagy, inflammation, and neuroprotection.

Both *in vitro* and *in vivo* experimental studies were included, whereas purely computational or clinical reports were cited only for contextual relevance. Priority was given to peer-reviewed studies with clear mechanistic endpoints and reproducible outcome measures to ensure methodological consistency. This approach provides a transparent overview of the molecular mechanisms and translational implications of naringenin in healthy and pathological aging.

## 2 Cellular and molecular framework of aging: mechanistic pathways and hallmark integration

This section outlines the cellular and molecular mechanisms that underlie aging and establishes the mechanistic framework for subsequent analysis of naringenin’s effects. Aging is a progressive, multifactorial process marked by a gradual reduction in the body’s ability to maintain physiological integrity. Over time, this decline affects tissue homeostasis, increases susceptibility to disease, and drives the development of a range of chronic conditions including cancer, diabetes, cardiovascular disorders, and neurodegenerative diseases (33). The biology of aging, once seen as a passive accumulation of damage, is now understood as an active, regulated phenomenon involving interconnected molecular pathways. Advances in molecular biology and genetics have revealed that aging is not solely stochastic but can be influenced by conserved genetic and biochemical mechanisms that control longevity and functional decline (34). The modern understanding of aging rests on a framework known as the “hallmarks of aging,” initially proposed to describe the fundamental cellular and molecular alterations that underpin the aging process. These hallmarks are grouped based on their roles in initiating, antagonizing, or integrating damage responses and have been instrumental in shaping geroscience research (35).

### 2.1 Early onset molecular pathologies in the aging process

At the core of aging lie mechanisms that directly contribute to molecular and cellular damage. One such mechanism is genomic instability, characterized by the accumulation of DNA mutations and chromosomal alterations over time. Endogenous factors like replication errors and ROS, as well as exogenous insults from radiation or environmental toxins, challenge genome fidelity. Although repair pathways exist, their efficacy diminishes with age, leading to accumulated damage that disrupts gene expression and cellular function (36, 37). Another hallmark is telomere shortening. Telomeres, the protective DNA-protein complexes at chromosome ends, erode with each cell division. When critically short, they trigger a DNA damage response, leading to cellular senescence. This process acts as a double-edged sword, suppressing tumorigenesis while accelerating tissue aging (38). Epigenetic modifications, including DNA methylation and histone changes, also evolve with age. These alterations modify chromatin structure and gene activity without changing the DNA sequence, leading to dysregulated expression of genes involved in inflammation, metabolism, and cell cycle regulation. Such epigenetic drift has been implicated in both aging and age-associated diseases (39). Loss of proteostasis, another primary hallmark, reflects the declining ability of cells to maintain protein quality. Misfolded proteins and damaged organelles accumulate due to overwhelmed or inefficient quality control systems like autophagy and the ubiquitin-proteasome system. These aggregates interfere with cellular function and can trigger inflammatory responses, further exacerbating age-related decline (40).

### 2.2 Paradoxical regulators of aging: from homeostasis to dysfunction

While primary hallmarks initiate cellular stress, antagonistic hallmarks represent the body’s early responses to such stress that become deleterious when sustained. Nutrient sensing pathways, particularly those involving insulin/IGF-1, mTOR, AMPK, and sirtuins, exemplify this. In youthful organisms, these pathways regulate growth and energy balance, but in aged tissues, their dysregulation promotes metabolic disorders. For instance, persistent activation of mTOR accelerates cellular aging, while its inhibition (e.g., by calorie restriction or rapamycin) has been shown to extend lifespan in various species (41, 42). Mitochondrial dysfunction is another central feature of aging. Mitochondria generate ATP through oxidative phosphorylation but also produce ROS as byproducts. In aged cells, mitochondrial efficiency declines, ROS production rises, and mitophagy becomes impaired. This feedback loop results in damage to mitochondrial DNA, lipids, and proteins, further reducing cellular energy capacity and increasing oxidative burden (43, 44). Cellular senescence, once a protective mechanism against malignant transformation, becomes harmful with age. Senescent cells stop dividing but remain metabolically active, secreting a range of pro-inflammatory cytokines, chemokines, and proteases collectively known as the SASP. Persistent accumulation of these cells impairs tissue regeneration, disrupts normal intercellular signaling, and contributes to chronic inflammation (45, 46).

### 2.3 Converging hallmarks of aging: from tissue exhaustion to inflammaging

When damage surpasses repair capacity, integrative hallmarks emerge. Stem cell exhaustion results from both intrinsic aging of stem cells and extrinsic alterations in their niches. Reduced regenerative capacity of aged stem cells impairs tissue repair, contributing to frailty and organ dysfunction (47). Simultaneously, aging alters the quality of communication between cells. Hormonal, neural, and inflammatory signals become dysregulated, disrupting coordination across tissues. These alterations in intercellular communication promote systemic inflammation, a phenomenon termed “inflammaging.” Low-grade, persistent inflammation without overt infection accelerates aging by enhancing oxidative stress, promoting senescence, and impairing immune surveillance (48).

### 2.4 Next-generation hallmarks: integrating inflammation, metabolism, and microbiota

Ongoing research has identified additional hallmarks. Impaired autophagy—essential for clearing damaged cellular components—has been increasingly recognized. With aging, autophagic flux declines, leading to the buildup of dysfunctional mitochondria and toxic protein aggregates, further stressing proteostasis and promoting inflammation (49). Alterations in the gut microbiome—specifically, loss of microbial diversity and beneficial species—also contribute to systemic inflammation and metabolic imbalance. This dysbiosis interacts with intestinal barrier dysfunction and immune dysregulation, highlighting the gut–brain–immune axis as a key player in aging (50). Further, lipid metabolism undergoes significant changes. Accumulation of sphingolipids, such as ceramides in muscle, impairs function and may drive sarcopenia. Dysregulated cholesterol handling within lysosomes supports the inflammatory phenotype of senescent cells and contributes to tissue dysfunction (51, 52). Hormonal fluctuations also play a role. Decreased levels of anabolic hormones (e.g., estrogen, testosterone, growth hormone) and increased levels of catabolic ones (e.g., cortisol) influence metabolism, bone density, and cognitive function. These shifts, together with body composition changes—such as increased fat mass and reduced muscle mass—raise the risk of insulin resistance, cardiovascular disease, and frailty (53–55). Finally, the role of oxidative stress remains central. While ROS are vital for cell signaling at physiological levels, their accumulation damages DNA, proteins, and lipids when unregulated. Telomeres, for instance, are particularly vulnerable to oxidative injury, linking redox imbalance to cellular aging and genomic instability.

Despite some controversy, the oxidative stress theory of aging continues to inform therapeutic strategies targeting antioxidant defenses (56, 57). The hallmarks of aging represent a dynamic network of interacting pathways. Genomic instability, telomere erosion, mitochondrial decline, and altered signaling do not act in isolation but reinforce one another, creating feedback loops that drive cellular dysfunction (58). Understanding these relationships is crucial for identifying interventions that promote healthy aging. Targeted approaches—ranging from senolytics and calorie restriction mimetics to microbiota modulation and proteostasis enhancers—offer promising avenues for delaying age-related decline and extending healthspan.

## 3 Mechanistic role of NAR in promoting synaptic plasticity and cognitive resilience

This section focuses on mechanistic findings describing how naringenin modulates oxidative, inflammatory, and neurotrophic pathways relevant to synaptic integrity.

As mentioned earlier, NAR, a flavanone predominantly found in citrus fruits like grapefruits and oranges, has emerged as a promising natural compound with neuroprotective and cognition-enhancing potential (59). It is primarily present either in its aglycone form or as glycosides such as naringin and narirutin (60). Owing to its unique molecular configuration, NAR readily interacts with cellular signaling systems implicated in oxidative stress, neuroinflammation, and protein aggregation, factors closely linked to synaptic dysfunction and cognitive decline (61). Despite its hydrophobic nature and limited water solubility, NAR has been shown to cross the blood-brain barrier, likely through passive diffusion or specific transporter-mediated processes (62). Once in the brain, it preferentially accumulates in regions critical to learning and memory. Preclinical studies have highlighted its antioxidant capacity, which is one of its core mechanisms of action. NAR effectively reduces intracellular ROS and boosts the activity of key antioxidant enzymes including SOD, CAT, and GPx (63, 64). These effects help preserve mitochondrial function and protect neurons from oxidative insults, a vital defense against early pathological changes in AD (65). In parallel, NAR attenuates neuroinflammatory signaling by suppressing pro-inflammatory cytokines such as TNF-α, IL-1β, and IL-6. It inhibits critical transcription factors including NF-κB and MAPK, thereby reducing glial activation and neuronal apoptosis. These anti-inflammatory effects have been linked to improvements in memory performance in both aging and AD models (66–69). Furthermore, NAR interferes with amyloidogenic pathways by downregulating the expression of APP and BACE1, contributing to decreased Aβ generation. Simultaneously, it promotes autophagic clearance of aggregated proteins and lowers GSK-3β activity, ultimately curbing tau hyperphosphorylation and preventing neurofibrillary tangle formation (70, 71). At the synaptic level, NAR has demonstrated the ability to enhance synaptic plasticity, a fundamental process underlying learning and memory. It positively modulates brain-derived neurotrophic factor (BDNF) signaling and upregulates its receptor TrkB, leading to activation of the downstream CREB pathway, an essential axis for long-term potentiation and synaptic remodeling.

Beyond its antioxidant and mitochondrial effects, naringenin appears to influence neurotrophin-mediated signaling cascades critical for synaptic plasticity. Experimental evidence suggests that naringenin enhances brain-derived neurotrophic factor (BDNF) and nerve growth factor (NGF) expression in neuronal and glial cultures exposed to oxidative or inflammatory stress. This effect is accompanied by activation of the TrkB–ERK–CREB axis, which governs dendritic remodeling and long-term potentiation. While direct receptor binding has not been demonstrated, naringenin’s ability to restore redox homeostasis and reduce NF-κB–mediated cytokine release likely facilitates neurotrophin signaling indirectly by maintaining neuronal excitability and transcriptional responsiveness (72–74). These interlinked pathways provide a mechanistic bridge between its antioxidant activity and synaptic resilience.

In animal studies, long-term administration of NAR restored spatial learning and motor coordination in aged rats, effects that correlated with elevated SIRT1 expression and reduced acetylated NF-κB in the hippocampus (75, 76). These findings suggest that NAR exerts its cognitive benefits by not only reducing neuroinflammation but also promoting transcriptional programs associated with neuronal survival and plasticity (77). Additionally, studies in vascular dementia models have revealed that NAR mitigates cognitive impairment by reducing hippocampal oxidative stress, enhancing the activity of SOD and GPx, and modulating cytokine profiles. Notably, it increases the levels of IL-10 and IL-4 while suppressing pro-inflammatory mediators (78, 79). At the molecular level, NAR significantly upregulates proteins essential to synaptic structure and function, including synaptophysin (SYP), postsynaptic density protein 95, and NMDA receptor subunits NR1 and NR2B (80).

Further support for its role in synaptic maintenance comes from observations that NAR enhances the expression of SYP, a marker critical for synaptic vesicle integrity and neurotransmission. This effect was evident in diabetic rat models, suggesting a broader neuroprotective role even beyond dementia (81). Mechanistically, NAR may restore synaptic integrity by influencing calcium/calmodulin-dependent protein kinase II (CaMKII) signaling. CaMKII is pivotal in regulating AMPA receptor phosphorylation, synaptic vesicle release, and dendritic spine formation. Through modulation of this pathway and inhibition of GSK-3β, NAR appears to prevent Aβ-induced synaptic deficits and support memory formation (82). Collectively, these findings position NAR as a multifunctional flavonoid that targets oxidative stress, inflammation, amyloidogenesis, and synaptic plasticity. Its influence on neurotrophic signaling and synaptic proteins suggests strong therapeutic potential for preventing or slowing cognitive decline in aging and neurodegenerative diseases. Together, these findings position naringenin as a modulator of redox and neurotrophic homeostasis, linking molecular protection to higher-order synaptic function.

## 4 Molecular and cellular hallmarks of aging targeted by NAR

Aging is marked by gradual functional decline driven by oxidative stress, mitochondrial dysfunction, and chronic low-grade inflammation. ROS damage essential biomolecules, while aged mitochondria lose efficiency and increase oxidative burden (83). Concurrently, persistent inflammation—known as “inflammaging”—arises from immune activation, senescent cells, and impaired autophagy, contributing to neurodegeneration and cardiovascular disease (84, 85). NAR, a flavonoid found in citrus fruits, shows potential in counteracting these aging processes. It enhances antioxidant defenses, supports mitochondrial function, and reduces inflammation. By targeting these core mechanisms, NAR may help preserve cellular health, delay aging, and lower the risk of age-related disorders. This section investigates its molecular actions across these interconnected pathways.

### 4.1 Oxidative stress and mitochondrial dysfunction

Aging is tightly linked to the gradual decline of cellular defense mechanisms and the increased production of ROS, which compromise mitochondrial function and damage macromolecules. Oxidative stress and mitochondrial impairment are not only central contributors to cellular senescence but also key drivers of age-related diseases such as neurodegeneration, intervertebral disc degeneration (IDD), and cardiovascular dysfunction. These interconnected phenomena result from an imbalance between ROS generation and antioxidant capacity, leading to disruption of redox homeostasis and bioenergetic failure (86, 87). Emerging evidence suggests that NAR, a flavonoid found in citrus fruits, can target these molecular events and exert protective effects by modulating oxidative damage and mitochondrial function in both neural and non-neural tissues.

To enhance readability and reduce textual redundancy, the following tables summarize the main preclinical models, signaling pathways, and outcomes describing naringenin’s mechanistic and functional effects. Together, Tables 1–3 provide a comparative overview of the SIRT1, Nrf2, FOXO, and MAPK axes across different experimental systems, highlighting the strength and consistency of current preclinical evidence.

**TABLE 1:** Comparative summary of key signaling pathways (SIRT1, Nrf2, FOXO, MAPK) modulated by naringenin across *in vitro* and *in vivo* preclinical models.

Model type	Inducer of aging/stress	Intervention (dose/route/duration)	Behavioral or phenotypic outcomes	Targeted pathways or molecular targets	Oxidative stress markers	Mitochondrial parameters	Key antioxidant genes/proteins	Distinctive mechanistic insight	References
Mouse (CNS, aging model)	d-Galactose (100 mg/kg)	Naringenin, 50 mg/kg, oral, 6 weeks	Improved locomotion and memory; reduced neuronal damage	PI3K/Akt/Nrf2 pathway	↓TBARS, ↑SOD, ↑CAT	Not assessed	↑HO-1, ↑NQO1	Enhances antioxidant defense via Nrf2 nuclear translocation	(88)
Nucleus pulposus cells *(in vitro*)	IL-1β (20 ng/mL)	Naringenin, 50 μM, 24–48 h	↓Senescence, ↑proliferation, ↓degeneration markers	IGFBP3 inhibition	↓ROS, ↑Gpx3, ↑Sod2, ↑Nfe2l2	Not assessed	↑Gpx3, ↑Sod2, ↑Nfe2l2	NAR inhibits IGFBP3, restoring redox balance and reducing degeneration	(89)
Mouse (brain aging model)	d-Galactose (100 mg/kg)	Naringin, 40/80 mg/kg, oral, 6 weeks	Improved spatial memory and cognition	Mitochondrial complex enzymes (I–III)	↓MDA, ↑SOD, ↑CAT	↑Complex I–III activity, ↑ATP, ↑membrane potential	Not specified	Naringin restores mitochondrial bioenergetics and antioxidant defense	(90)
Mouse (heart aging model)	Natural aging (12-month-old mice)	Naringenin, 100 mg/kg/day, oral, 6 months	Reduced fibrosis, improved cardiac function	SIRT1 activation	↓ROS (DHE staining)	↑Citrate synthase activity, ↑mitochondrial membrane potential	↑SIRT1 mRNA and protein	Mimics resveratrol; long-term SIRT1 activation restores redox homeostasis	(91)
Rat (1-year-old heart and mitochondria)	Natural aging + ischemia/reperfusion	Naringenin, 10–100 μM (ex vivo)	Reduced infarct size; preserved cardiac function	mitoBK channel activation	↓ROS (cardiac tissue)	↑Mitochondrial membrane depolarization (functional mitoBK opening)	Not specified	NAR activates mitoBK to improve mitochondrial K+ flux and cardioprotection	(92)

PI3K/Akt/Nrf2, Phosphatidylinositol 3-kinase/Protein kinase B/Nuclear factor erythroid 2-related factor *2* signaling pathway; TBARS, Thiobarbituric Acid Reactive Substances; SOD, Superoxide Dismutase; CAT, Catalase*;* HO-1, Heme Oxygenase-1; NQO1, NAD(P)H Quinone Dehydrogenase 1; IGFBP3 inhibition, Inhibition of Insulin-like Growth Factor Binding Protein 3; Gpx3, Glutathione Peroxidase 3; Nfe2l2, Nuclear Factor, Erythroid 2 Like 2 (gene encoding Nrf2); MDA, Decreased Malondialdehyde; Complex I–III activity, activity of mitochondrial respiratory complexes I, II, and III; ATP, Adenosine Triphosphate; SIRT1 activation, Activation of Sirtuin 1; ROS (DHE staining), Reactive Oxygen Species levels as assessed by Dihydroethidium (DHE); IL-1β, Interleukin-1 beta.

**TABLE 2:** Experimental models, interventions, and outcomes demonstrating naringenin’s effects on oxidative, mitochondrial, and autophagic pathways in both *in vitro* and *in vivo* systems.

Model system	Aging induction method	Compound	Dose and route	Key molecular targets/pathways	Functional outcomes	Tissue/organ assessed	Mechanisms affected	Experimental duration	Histology/ imaging findings	Conclusion/ implication	References
*C. elegans*, Middle-aged mice	Natural aging	Naringenin	100 μM (*C. elegans*), 100 mg/kg/day orally (mice)	SIRT1, citrate synthase (CS), cytochrome c oxidase (CcO), p16, Il-6, Il-18	↑ Lifespan and motility (worms), ↑ mitochondrial enzymes (mice), ↓ senescence markers	Whole organism (worms), brain (mice)	Mitochondrial activity, oxidative stress, inflammation, senescence	6 months (mice)	Reduced p16/Il-6 mRNA, increased locomotion in worms	Delays brain aging; promotes mitochondrial and antioxidant function	(96)
D-galactose-induced aging rats	D-galactose injection	Naringenin	50 and 100 mg/kg/day orally	TLR4, MyD88, p38 MAPK, NF-κB, TNF-α, IL-1β	↓ Inflammation, ↑ cognition (Morris Water Maze)	Hippocampus	Inflammation, cognitive impairment	8 weeks	↓ Neuronal loss, ↓ microglial activation	Attenuates neuroinflammation via TLR4/MyD88 inhibition	(97)
Rats	D-galactose (150 mg/kg, subcutaneous injection)	Naringin	100 mg/kg, oral (gavage)	TLR4, NF-κB, TNF-α, IL-1β, IL-6, MCP-1, GRP78, CHOP, ATF6, BDNF, NGF, MDA, GSH-Px	↑ Cognitive performance (MWM, NORT, fear conditioning); ↑ neurotrophic factors; ↓ inflammatory cytokines, ↓ oxidative and ER stress markers	Hippocampus	Neuroinflammation, oxidative stress, ER stress, neurotrophic support, TLR4/NF-κB signaling	Not specified (chronic protocol until endpoint)	↓ Neuronal and vertebral damage in hippocampus; ↓ cytoplasmic vacuolization and karyopyknosis	Naringin ameliorates cognitive dysfunction and hippocampal pathology by inhibiting TLR4/NF-κB and ER stress; promotes neurotrophic support	(98)
SAMP8 mice (senescence-accelerated mouse model)	High-fat diet (HFD)	Naringenin	0.2% Dietary administration, oral	Aβ production, tau phosphorylation, oxidative stress markers, neuroinflammatory markers	↑ Spatial learning and memory (Barnes Maze, Morris Water Maze); ↓ Aβ, tau-P, oxidative stress, inflammation	Brain	Amyloid pathology, tau pathology, redox imbalance, neuroinflammation	12 weeks	Not explicitly detailed; improvement in biochemical and cognitive markers indicates neuroprotection	Naringenin ameliorates cognitive deficits in HFD-fed aging SAMP8 mice by targeting multiple AD-related pathologies	(99)
H9c2 myocardial cells (*in vitro*)	H_2_O_2_ exposure (60 μM for 3 h)	Naringenin	Various concentrations in culture medium (*in vitro*)	ROS, p16, p21, mitochondrial CRC, ERβ, VDR, estrogenic signaling	Reduced senescence (β-gal staining), decreased DNA damage, enhanced mitochondrial function	Cardiomyocytes (*in vitro* model)	Oxidative stress, mitochondrial metabolism, calcium homeostasis, estrogenic gene regulation	72 h post-treatment	Reduced X-gal staining, improved CRC capacity, decreased ROS, restored gene expression	Naringenin alleviates H_2_O_2_-induced senescence in myocardial cells via antioxidative, mitochondrial, and estrogenic mechanisms	(30)

Models are stratified according to experimental design: *in vitro* studies (neuronal, glial, or fibroblast cultures) and *in vivo* studies (rodent or nematode models) were included to illustrate cross-system consistency. IL-6, Interleukin-6; SIRT1, Sirtuin-1; TLR4, Toll-like receptor 4; MyD88, Myeloid differentiation primary response 88; p38 MAPK, p38 mitogen-activated protein kinase; MCP-1, Monocyte chemoattractant protein-1; GRP78, Glucose-regulated protein 78; CHOP, C/EBP homologous protein; ATF6, Activating transcription factor 6; BDNF, Brain-derived neurotrophic factor; NGF, Nerve growth factor; MDA, Malondialdehyde; GSH-Px, Glutathione peroxidase; MWM, Morris Water Maze; NORT, Novel Object Recognition Test; ER stress, Endoplasmic Reticulum Stress; CRC capacity, Calcium Retention Capacity of mitochondria; H_2_O_2_, Hydrogen Peroxide.

**TABLE 3:** Functional and behavioral outcomes observed in aging and neurodegenerative models following naringenin intervention, emphasizing translational relevance.

Model type	Inducer of aging/stress	Intervention (dose/route/duration)	Behavioral or phenotypic outcomes	Targeted pathways/molecular targets	Oxidative-stress markers	Mitochondrial parameters	Key antioxidant or trophic genes/proteins	Distinctive mechanistic insight	References
Aging mouse model	d-Galactose (100 mg/kg, s.c.)	NAR 100 mg/kg oral × 8 weeks	Improved cognition (MWM, open-field); reduced hippocampal damage	PI3K/Akt→ Nrf2/HO-1/NQO1 cascade	↓ TBARS, ↑ SOD, ↑ CAT	Not directly measured	↑ HO-1, ↑ NQO1, ↑ Nrf2	Enhances antioxidant defenses through Nrf2 activation and downstream gene up-regulation	(88)
Aging mouse retina	Physiological aging	NAR 100 mg/kg oral daily × 6 months (long-term)	Preserved retinal structure; improved ERG responses	MFN2 ↑ / DRP1 ↓ / autophagy-related proteins	Not specified	↑ MFN2, ↓ DRP1 → improved fusion–fission balance	↑ LC3, ↑ PINK1 (trends reported)	Restores mitochondrial dynamics and autophagy in aged retina	(110)
Aging neural stem cells (NSCs) and mice	Replicative senescence (23-month NSCs) / physiological aging (12-month mice)	NAR 6.8 μg/mL (*in vitro*, 72 h) + 20 mg/kg i.v. (*in vivo*, daily × 4 weeks)	↑ NSC proliferation, ↓ p16^Ink4a^, ↑ spatial memory and neurogenesis	TNF-α↓, Ki-67 ↑, MAP2 ↑, telomerase ↑	↓ ROS in NSCs	Not assessed *in vivo*	↑ Ki-67, ↑ MAP2, ↑ telomerase	Promotes NSC rejuvenation and neurogenesis through TNF-α modulation and redox regulation	(111)

Aβ, amyloid-β; p-tau, phosphorylated tau; CAT, catalase; ERG, electroretinogram; HO-1, heme oxygenase-1; MAP2, microtubule-associated protein-2; MFN2, mitofusin-2; MWM, Morris Water Maze; NQO1, NAD(P)H quinone dehydrogenase 1; Nrf2, nuclear factor erythroid 2–related factor 2; NSC, neural stem cell; ROS, reactive oxygen species; SOD, superoxide dismutase; TNF-α, tumor necrosis factor-α.

In a mouse model of brain aging induced by chronic d-galactose exposure, Zhang et al. (88) demonstrated that NAR significantly reduced behavioral impairments and oxidative stress levels. In this model, long-term administration of d-galactose led to cognitive dysfunction, increased lipid peroxidation, and diminished antioxidant defenses. Treatment with NAR activated the PI3K/Akt pathway, which facilitated the nuclear translocation of Nrf2—a key regulator of antioxidant gene expression. This molecular event was accompanied by elevated expression of downstream antioxidant enzymes, including HO-1 and NAD(P)H quinone dehydrogenase 1 (NQO1).

Enzymatic assays confirmed that NAR restored the activity of key antioxidant enzymes such as SOD and catalase (CAT), and reduced thiobarbituric acid reactive substances (TBARS), indicative of lower lipid peroxidation. These results highlight the ability of NAR to enhance endogenous antioxidant capacity and mitigate oxidative damage, thus preserving neuronal structure and function (88). Beyond its effects in neural tissue, NAR also demonstrates protective potential in the context of intervertebral disc aging. Tang et al. (89) investigated the role of NAR in human nucleus pulposus cells (NPCs) subjected to IL-1β-induced degeneration—a model relevant to IDD. In these cells, oxidative stress was a prominent pathological driver, as indicated by elevated intracellular ROS levels and reduced expression of antioxidant defense genes. Treatment with NAR significantly lowered ROS accumulation and upregulated the transcription of genes encoding antioxidative enzymes, including Gpx3, SOD2, and Nrf2. These molecular changes were associated with the attenuation of senescence markers and a reduction in catabolic gene expression. Importantly, Tang et al. (89) identified insulin-like growth factor binding protein 3 (IGFBP3) as a molecular mediator that interferes with NAR’s protective effects. The presence of exogenous IGFBP3 reversed NAR-mediated improvements, reinforcing the importance of this signaling axis in ROS-driven disc degeneration. This study further confirms that ROS-induced damage and altered redox signaling are central to age-associated tissue degeneration, and that NAR may intervene through both antioxidant activity and IGFBP3-dependent regulation (89). Kumar et al. (90) explored the therapeutic potential of naringin, the glycoside precursor of NAR, in a similar d-galactose-induced model of cognitive decline. Aging induced by d-galactose led to impaired performance in spatial memory tasks and a significant decline in mitochondrial complex I, II, and III activities. These alterations were accompanied by elevated oxidative stress markers and compromised energy metabolism. Treatment with naringin not only improved behavioral performance but also restored the activities of the mitochondrial respiratory chain enzymes, suggesting that flavonoids like naringin or NAR can preserve mitochondrial bioenergetics in the context of oxidative insult.

This restoration of mitochondrial function may be closely tied to their antioxidant properties, highlighting the capacity of these flavonoids to sustain both redox equilibrium and ATP generation in aging neurons (90). Aging-related deterioration of cardiac function is also tightly associated with mitochondrial dysfunction and oxidative stress. In a comprehensive study by Testai et al. (91), NAR was found to exert cardioprotective effects in aged mice by activating SIRT1, a NAD+-dependent deacetylase involved in cellular stress resistance and mitochondrial health. In 12-month-old mice, cardiac expression of SIRT1 was significantly reduced compared to younger controls, whereas chronic NAR treatment restored SIRT1 expression at both protein and mRNA levels. In parallel, DHE staining of heart tissue revealed elevated ROS levels in aged mice, which were markedly diminished following NAR administration. These improvements were associated with decreased expression of pro-inflammatory cytokines (TNF-α and IL-6), reduction in fibrotic remodeling, and enhanced activity of mitochondrial citrate synthase. Importantly, NAR also stabilized mitochondrial membrane potential, suggesting a direct role in preserving mitochondrial integrity under age-related stress. Through SIRT1 activation and ROS suppression, NAR thus appears to support mitochondrial function and attenuate oxidative burden in the aging heart (91).

Further insights into the mitochondrial effects of NAR were provided by Testai et al. (92) in a follow-up study involving ischemia/reperfusion (I/R) injury in aged rat hearts. The researchers focused on the mitochondrial large-conductance calcium-activated potassium (mitoBK) channels, which are crucial regulators of mitochondrial membrane potential and cardioprotection. In aged rats, NAR significantly reduced infarct size and protected cardiac tissue from I/R-induced damage. These effects were abolished by paxilline, a selective blocker of mitoBK channels, confirming that NAR’s protective action was channel-dependent. Isolated mitochondria from aged rats displayed a moderately depolarized membrane potential compared to younger animals. NAR induced a concentration-dependent depolarization of the mitochondrial membrane, which was reversed by paxilline, suggesting that mitoBK channel activation was responsible for the observed effects. This finding underscores NAR’s ability to fine-tune mitochondrial electrophysiology and its potential to support mitochondrial resilience during age-related cardiac stress (92). In addition to these organ-specific findings, the collective data from the five studies indicate that NAR operates through a common set of mechanisms that converge on mitochondrial stabilization and oxidative stress reduction. Activation of transcriptional regulators such as Nrf2 and SIRT1 enables NAR to modulate a wide array of downstream targets involved in redox balance, inflammation, and energy metabolism (88, 91). By enhancing mitochondrial complex activities, preserving membrane potential, and reducing excessive ROS, NAR protects cells from entering a pro-senescent and dysfunctional state. These mechanisms are observed not only in neurons and cardiomyocytes (92) but also in structural cells like NPCs (89), suggesting a systemic relevance of NAR across tissues commonly affected by aging. Moreover, these findings reinforce the notion that age-related mitochondrial dysfunction is not solely a consequence of accumulated damage, but also a modifiable process subject to intervention through exogenous agents (91). NAR appears capable of resetting redox balance and restoring mitochondrial capacity even in advanced stages of aging. The studies by Zhang et al. (88), Tang et al. (89), and Testai et al. (91, 92) demonstrate that targeting oxidative stress and mitochondrial pathways using natural flavonoids can yield tangible benefits across distinct age-sensitive systems.

It is also notable that NAR’s action is both direct and indirect: It scavenges ROS, but also regulates gene expression via redox-sensitive transcription factors. For instance, Nrf2 activation by NAR not only boosts antioxidant defenses but also supports mitochondrial quality control through autophagic mechanisms. Similarly, SIRT1 activation contributes to mitochondrial biogenesis and suppression of inflammatory signaling. In the heart, this manifests as reduced cytokine expression and fibrosis; in the brain, as preserved neuronal survival and function; and in the spine, as maintenance of disc integrity (Table 1). Taken together, these studies strongly support the integration of NAR into strategies aimed at mitigating mitochondrial dysfunction and oxidative damage associated with aging. The ability of NAR to engage multiple protective pathways—ranging from PI3K/Akt and Nrf2 activation to SIRT1 stimulation and mitoBK channel opening—positions it as a multi-target agent with broad therapeutic relevance. Its low toxicity and natural dietary origin further enhance its appeal as a nutraceutical compound for promoting healthy aging.

### 4.2 Inflammation and SASP modulation

Chronic, low-grade inflammation is a hallmark of biological aging and plays a crucial role in the pathophysiology of numerous age-related diseases, including neurodegenerative, cardiovascular, metabolic, and musculoskeletal disorders (93). One of the core drivers of this sustained inflammatory milieu is the SASP, a process wherein senescent cells, having exited the cell cycle, continue to secrete a broad array of pro-inflammatory cytokines, chemokines, growth factors, and matrix-remodeling enzymes. The SASP not only disrupts tissue homeostasis but also amplifies the spread of senescence to neighboring cells through paracrine signaling, thereby exacerbating the decline in tissue function and regenerative capacity (94, 95). Growing preclinical evidence suggests that plant-derived flavonoids such as NAR and naringin may counteract this pro-inflammatory state by modulating SASP-related molecular pathways across a variety of tissues. In a pivotal study conducted by Piragine et al. (96), NAR supplementation in middle-aged mice over a period of 6 months was associated with a significant upregulation of genes involved in anti-inflammatory and antioxidant defense mechanisms. Specifically, there was an observed increase in the expression of Il-6 and Il-18, which may initially seem paradoxical given their traditional classification as pro-inflammatory cytokines. However, in certain physiological contexts, especially during aging, IL-6 also plays roles in maintaining tissue integrity and immune surveillance (96). Moreover, genes associated with oxidative stress resistance and longevity, such as SIRT1, Forkhead box O3 (Foxo3), and nuclear factor erythroid 2–related factor 2 (Nrf2), were also elevated in response to NAR. Importantly, the study noted that the age-related rise in the expression of p16Ink4a, a cyclin-dependent kinase inhibitor and senescence marker, was attenuated in the NAR -treated group, indicating a potential suppression of the senescence program and SASP induction (96). Further supporting the anti-inflammatory effects of naringin, Salama et al. (97) demonstrated its neuroprotective potential in a D-galactose-induced aging model in rats. Behavioral assessments showed improved learning and memory, suggesting cognitive restoration. These improvements were underpinned by molecular data showing reduced hippocampal expression of tumor necrosis factor-alpha (TNF-α) and nuclear factor kappa-light-chain-enhancer of activated B cells (NF-κB), both of which are central mediators of SASP and chronic inflammation. Naringin also restored levels of neurotrophic factors, including peroxisome proliferator-activated receptor gamma coactivator 1-alpha (PGC-1α) and neurotrophin-3 (NT-3), suggesting enhanced mitochondrial biogenesis and neuronal resilience. Additionally, reductions in advanced glycation end products (AGEs) and glial fibrillary acidic protein (GFAP) implied decreased oxidative stress and astrocyte activation, respectively, both of which are prominent in inflammaging (97).

Expanding on this, Dai et al. (98) investigated the effects of naringin in another D-galactose-induced cognitive aging model, focusing on the TLR4/NF-κB signaling axis. Toll-like receptor 4 (TLR4) is a pattern recognition receptor that activates NF-κB in response to endogenous danger signals, thereby triggering a cascade of inflammatory gene expression, including IL-1β, IL-6, and monocyte chemoattractant protein-1 (MCP-1), all key SASP components. Naringin administration significantly suppressed these pro-inflammatory cytokines in the hippocampus. Interestingly, the study also highlighted a reduction in endoplasmic reticulum (ER) stress markers, including GRP78, CHOP, and ATF6, suggesting that naringin not only dampens inflammatory signaling but also alleviates proteostasis disruptions. ER stress is increasingly recognized as a contributor to SASP initiation, particularly in metabolically active tissues such as the brain. In conjunction with these anti-inflammatory effects, Dai et al. (98) reported that naringin enhanced the activities of antioxidant enzymes like (GPx), CAT, and SOD, while decreasing lipid peroxidation marker malondialdehyde (MDA). The redox balance maintained by these enzymes is essential to prevent ROS-induced activation of pro-inflammatory transcription factors, and their modulation contributes to reduced SASP activity. Furthermore, the treatment group showed elevated levels of BDNF and nerve growth factor (NGF), which are essential for maintaining synaptic plasticity and neuronal survival, both of which are impaired in the context of neuroinflammation (98). Zhou et al. (99) contributed additional insights in a high-fat diet-induced aging model in mice, demonstrating that dietary supplementation with NAR ameliorated cognitive impairments and reduced neuroinflammatory markers. NAR administration led to a significant reduction in the accumulation of Aβ plaques and phosphorylated tau, which are associated with neuroinflammation and microglial activation in Alzheimer’s disease. Although not direct SASP components, these neuropathological features are linked with the senescence-related inflammatory milieu. Hence, the attenuation of Aβ and tau pathology reflects NAR’s capacity to mitigate neurodegenerative processes that are potentiated by chronic inflammation and SASP (99).

Recent reviews and experimental reports through 2024–2025 reinforce and extend naringenin’s multi-modal effects in AD models. A recent focused review synthesizes advances in epigenetic signatures, gut microbiota interactions, and delivery strategies for naringenin in Alzheimer’s disease, concluding that while mechanistic evidence is growing, translational hurdles (formulation, CNS exposure, and standardized endpoints) remain to be addressed (100). Notably, a 2024 preclinical study reported that naringenin reduces Aβ burden and neuroinflammation while improving synaptic markers, supporting the view that naringenin acts on both amyloidogenic and neuroinflammatory axes relevant to AD pathophysiology (101).

Noteworthy, many preclinical reports use either naringin (the glycoside) or naringenin (the aglycone), and these compounds differ substantially in pharmacokinetics and CNS exposure. Naringin is more hydrophilic and is often subject to intestinal hydrolysis and first-pass metabolism; its tissue distribution is biased toward liver and bile unless converted to the aglycone, whereas naringenin (aglycone) is relatively more lipophilic and may achieve higher membrane and CNS partitioning under some conditions (24). Recent studies also show that naringenin’s BBB-protective and CNS effects are formulation- and metabolism-dependent, emphasizing that results obtained with naringin cannot be assumed for naringenin (and vice versa) without considering dose, route, and metabolic conversion (102).

Da Pozzo et al. (30) explored NAR’s role beyond the central nervous system, using a cardiac cell model of oxidative stress-induced senescence. Treatment with NAR reduced the expression of classical senescence markers p16 and p21 in H9c2 cardiomyoblasts and restored the cells’ ability to proliferate, as evidenced by normalization of cell cycle progression. These changes were accompanied by decreased levels of ROS and DNA strand breaks, both of which are upstream triggers of SASP activation. Moreover, NAR significantly improved mitochondrial parameters, including membrane potential and calcium retention capacity, both of which are essential for maintaining bioenergetic efficiency and preventing mitochondrial-derived ROS generation. In addition, Da Pozzo et al. (30) highlighted NAR’s ability to modulate hormone receptor signaling in aging cardiomyocytes. Specifically, NAR upregulated estrogen receptor beta (ERβ) and vitamin D receptor (VDR), both of which have been implicated in the suppression of inflammatory pathways and promotion of mitochondrial biogenesis. Given that postmenopausal estrogen deficiency is linked with increased cardiovascular senescence and inflammation, this hormonal regulation offers a novel sex-specific dimension to NAR’s anti-SASP effects (30).

#### 4.2.1 Modulation of pro-inflammatory signaling

Both compounds consistently downregulate NF-κB and TLR4 expression and activity, leading to decreased production of canonical SASP factors like IL-1β, TNF-α, and IL-6. This has been validated in both central and peripheral tissues.

#### 4.2.2 Reduction of oxidative and ER stress

Through upregulation of antioxidant defenses and attenuation of ER stress markers, these flavonoids help restore redox and proteostatic homeostasis, thereby preventing stress-induced SASP initiation.

#### 4.2.3 Suppression of senescence markers

Studies report consistent reductions in p16, p21, and SA-β-gal expression across neuronal and cardiac models, reflecting a direct impact on the senescence program.

#### 4.2.4 Restoration of mitochondrial and trophic support

Enhancements in mitochondrial function, redox capacity, and neurotrophin levels such as BDNF and NGF promote resilience against age-associated functional decline.

#### 4.2.5 Hormonal and epigenetic modulation

The upregulation of ERβ, VDR, and longevity-associated transcription factors like SIRT1 and Foxo3 suggests that these flavonoids exert multi-level regulatory control, possibly including epigenetic reprogramming.

Importantly, the protective actions of NAR and naringin were consistent across a range of experimental models, including oxidative, diet-induced, and replicative senescence, and spanned multiple organ systems. These compounds thus offer a versatile and integrative approach to targeting inflammaging and SASP, with promising implications for aging interventions.

In summary, NAR and naringin demonstrate significant promise as natural senotherapeutics capable of mitigating inflammaging and suppressing the harmful cascade of SASP activation. Their ability to modulate inflammatory cytokines, senescence markers, oxidative stress, and mitochondrial function positions them as potent multi-target compounds in the field of aging research. Given their favorable safety profiles and presence in common dietary sources, these flavonoids hold strong translational potential. Future studies are needed to validate these effects in human subjects and to explore synergistic combinations with other anti-aging strategies for comprehensive management of age-related inflammation and functional decline (Table 2).

While several natural antioxidants, including quercetin, resveratrol, and epigallocatechin gallate, exert neuroprotective actions through overlapping redox and mitochondrial pathways, naringenin presents a distinct biochemical and pharmacokinetic profile. Its relatively higher lipophilicity and aglycone structure facilitate membrane interaction and moderate blood–brain barrier permeability, while maintaining balanced antioxidant capacity without excessive pro-oxidant stress (62). In contrast to polyhydroxylated flavonoids such as quercetin, naringenin exhibits milder autoxidation and greater metabolic stability, which may partly explain its favorable tolerability in chronic administration models (103). Together, these comparative features situate naringenin within a broader class of nutraceutical compounds acting through convergent mitochondrial and redox mechanisms (104).

## 5 Lifespan and healthspan enhancement by NAR: a multifaceted intervention against aging

**FIGURE 1:**
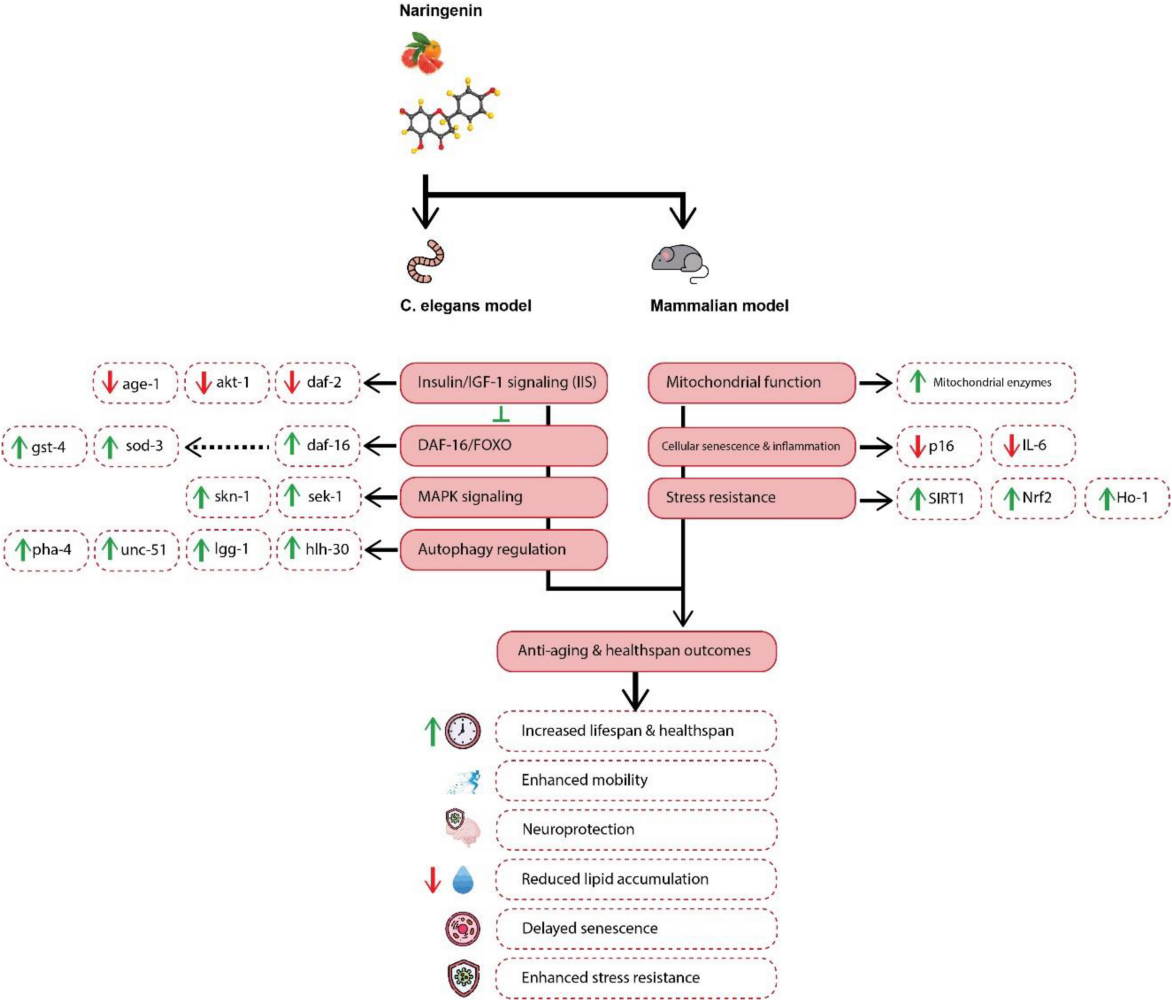
Naringenin and naringin extend lifespan and healthspan through conserved molecular pathways in *Caenorhabditis elegans* and Mammalian models. Naringenin and its glycoside naringin exhibit broad anti-aging effects in both *C. elegans* and mammalian models. In *C. elegans*, they extend lifespan, enhance mobility, and reduce aging markers through activation of conserved pathways such as insulin/IGF-1 (IIS), DAF-16/FOXO, and MAPK. These compounds boost antioxidant defenses (sod-3, gst-4), promote autophagy (hlh-30, lgg-1), and regulate lipid metabolism. Their neuroprotective role is evident in Alzheimer’s and Parkinson’s disease models, where naringin reduces α-synuclein aggregation and preserves neurons. In mammals, naringenin improves mitochondrial enzyme activity and lowers expression of aging markers (p16^Ink4a^, IL-6), while upregulating Sirt1, Nrf2, and Ho-1. These findings position naringenin/naringin as promising geroprotective agents.

NAR a naturally occurring citrus flavanone, has gained considerable attention as a promising bioactive compound with anti-aging properties. Recent studies using the nematode *Caenorhabditis elegans* (*C. elegans*), a well-established model in aging research due to its short lifespan and genetic similarity to humans in longevity-related pathways (105), have provided compelling evidence for the lifespan- and healthspan-extending effects of NAR and its glycoside derivative, naringin. Across multiple investigations, NAR has demonstrated the capacity to modulate key signaling pathways—including DAF-16/FOXO, insulin/IGF-1 signaling (IIS), MAPK, autophagy regulation, and lipid metabolism—thereby delaying senescence and improving organismal vitality (Figure 1). Piragine et al. (96) demonstrated that NAR (100 μM) extended lifespan and improved mobility in aging *C. elegans*, indicating enhanced healthspan. In middle-aged mice, chronic treatment (100 mg/kg) increased brain mitochondrial enzyme activity, including citrate synthase and cytochrome c oxidase. These findings suggest that NAR promotes longevity and preserves neuromuscular and mitochondrial function, highlighting its conserved anti-aging potential across species. Beyond the phenotypic observations, (96) also examined molecular markers of cellular senescence and inflammation. Notably, in murine brain tissues, NAR treatment counteracted the age-related increase in p16 expression, a widely accepted marker of cellular aging, as well as IL-6, a pro-inflammatory cytokine associated with SASP. Upregulation of SIRT1, a key regulator of longevity and stress resistance, alongside antioxidant genes such as Nrf2 and Ho-1, was also observed. Together, these results suggest that NAR exerts multi-target protective effects, spanning metabolic support, antioxidant defense, and suppression of senescence signaling. Although these mechanisms were elucidated in mammalian systems, the foundational evidence in *C. elegans* validates the evolutionary conservation of these processes (96).

Building upon these findings, Zhu et al. (106) conducted an in-depth analysis of naringin—the glycosylated form of NAR—and demonstrated its significant lifespan-prolonging effect in *C. elegans*, predominantly through the DAF-16 transcription factor. DAF-16, a member of the FOXO family, is central to longevity regulation in *C. elegans* and is negatively regulated by the IIS pathway. Zhu et al. showed that naringin enhanced stress resilience, reduced oxidative damage, and delayed the onset of aging-related pathologies. Importantly, the lifespan extension effect of naringin was abolished in daf-16 null mutants, indicating that DAF-16 activation is essential for naringin’s pro-longevity action (106). In addition to general aging models, Zhu et al. (106) utilized disease-specific *C. elegans* strains that mimic aspects of human neurodegenerative disorders, including AD’s and PD. Treatment with 50 μM naringin significantly reduced α-synuclein aggregation in PD models and improved motor function, indicating neuroprotective properties. Similarly, in worms exposed to 6-hydroxydopamine, naringin preserved dopaminergic neuron integrity, with effects comparable to standard anti-Parkinsonian treatments such as levodopa. These findings highlight the therapeutic potential of naringin not only in general aging but also in mitigating age-related neurodegenerative processes, mediated again through DAF-16–associated stress response pathways (106). Further mechanistic insights were provided by Ge et al. (107), who focused on the antioxidant and anti-aging activities of NAR in *C. elegans*, emphasizing its modulation of the IIS and MAPK signaling cascades. Following treatment with NAR under both normal and oxidative stress conditions, worms displayed enhanced lifespan, improved mobility, and reduced aging pigments, notably lipofuscin accumulation. Gene expression profiling revealed upregulation of daf-16, sek-1, and skn-1, while components of the IIS pathway such as daf-2, age-1, and akt-1 were downregulated. These transcriptional changes led to the activation of downstream genes involved in oxidative defense, including sod-3, ctl-1, and gst-4. These data suggest that NAR promotes longevity by enhancing cellular stress resistance and reducing ROS accumulation.

The findings from Ge et al. (107) also underscore the importance of MAPK signaling in the regulation of aging. The upregulation of sek-1, a MAPKK that mediates stress responses, and skn-1, a homolog of mammalian Nrf2, indicates that NAR may exert its antioxidant effect through a dual mechanism involving both IIS suppression and MAPK pathway activation. Molecular docking studies conducted in the same study further supported direct interactions of NAR with key signaling proteins, offering a structural basis for its functional effects (107). In a complementary investigation, Guo et al. (108) examined the anti-aging and anti-obesity effects of naringin in a high-glucose-induced *C. elegans* model, which mimics aspects of metabolic aging. Treatment with 25 μM naringin resulted in a roughly 24% extension of mean lifespan and an 11% increase in fast movement span, reflecting both lifespan and healthspan improvements. Notably, naringin significantly reduced lipid accumulation in worms, as visualized through Oil Red O staining. This fat-lowering effect was accompanied by increased expression of autophagy-related genes, including hlh-30, lgg-1, unc-51, and pha-4, which are central regulators of autophagic flux and cellular maintenance. Guo et al. (108) further verified the mechanistic relevance of autophagy by employing genetic knockdowns. The beneficial effects of naringin were completely abrogated in hlh-30 mutants and following RNAi-mediated inhibition of key autophagy genes, confirming that naringin’s actions are dependent on autophagic activity. This suggests that the compound not only improves lipid metabolism but also maintains cellular quality control via enhanced autophagic degradation. Transcriptome analysis reinforced these observations by identifying upregulation of longevity pathways, as well as TGF-β and Wnt signaling axes. These data extend the understanding of naringin’s bioactivity to encompass nutrient-sensing and metabolic homeostasis mechanisms, in addition to classical stress-resistance pathways.

Taken together, the collective evidence from these studies presents a coherent picture of NAR and naringin as multifaceted modulators of aging. Across diverse *C. elegans* models—ranging from normal aging, oxidative stress, metabolic dysfunction, to neurodegeneration—these compounds consistently extend lifespan and improve physiological function. The mechanistic underpinnings are rooted in the activation of evolutionarily conserved pathways, including DAF-16/FOXO (106), IIS/MAPK signaling (107), autophagy induction, and lipid metabolism regulation (108). Notably, each pathway contributes to different aspects of the aging phenotype, such as stress resistance, mitochondrial maintenance, fat storage, and proteostasis, enabling a broad-spectrum protective effect. Moreover, the translational potential of these findings is supported by Piragine et al. (96) work in mammalian models, which highlights NAR’s ability to modulate mitochondrial enzymes and senescence markers without inducing toxicity. This suggests that long-term dietary supplementation of NAR or naringin might offer a feasible strategy for delaying age-related decline in humans. Future research is warranted to explore pharmacokinetics, optimal dosing, and synergistic interactions with other nutraceuticals in higher organisms. In conclusion, NAR and its derivative naringin represent promising natural compounds with the ability to delay aging and extend lifespan through diverse but interconnected molecular pathways. The consistent effects observed in *C. elegans* models, coupled with preliminary success in vertebrates, support the potential of these flavonoids as geroprotective agents. Continued investigation into their mechanisms and clinical applicability could pave the way for their integration into anti-aging therapeutic strategies.

## 6 Multifaceted neuroprotective actions of naringenin against brain aging and proteinopathies

Here, the emphasis shifts from mechanistic insight to translational relevance, summarizing how preclinical models demonstrate functional and behavioral outcomes of naringenin treatment.

Aging is closely associated with the progressive decline of cognitive and motor function, as well as increased vulnerability to neurodegenerative diseases such as AD and PD (109). Recent studies have shown that NAR, a naturally occurring flavonoid found in citrus fruits, exhibits strong neuroprotective potential across multiple models of brain aging and neurological impairment. Experimental evidence from invertebrate and mammalian models suggests that NAR can counteract oxidative stress, modulate neuroinflammatory cascades, enhance neurotrophic signaling, and reduce pathological protein aggregation (Table 3).

### 6.1 Behavioral and biochemical improvements in aging brain models

In a murine model of brain aging induced by d-galactose, Zhang et al. (88) demonstrated that NAR significantly attenuated behavioral abnormalities. Mice exposed to d-galactose exhibited marked deficits in locomotor activity and memory, as evaluated by open field and Morris Water Maze tests. NAR treatment reversed these behavioral changes, suggesting preserved neuronal function. Histological analyses further revealed that NAR reduced neuronal loss and structural degeneration in the hippocampus. Mechanistically, its benefits were linked to activation of the PI3K/Akt pathway, which led to nuclear translocation of Nrf2 and subsequent induction of antioxidant enzymes such as HO-1 and NQO1. Enhanced levels of SOD and CAT, along with reduced lipid peroxidation products, confirmed the antioxidative role of NAR in the aging brain (88). Parallel findings by Salama et al. (97) in another d-galactose-induced aging model supported the cognitive-enhancing properties of NAR, specifically its glycoside form, naringin. Their study showed that naringin not only improved object recognition, Y-maze alternation behavior, and locomotor activity, but also modulated mitochondrial function via upregulation of AMPK, LKB1, and PGC1-α. These markers are critical regulators of energy metabolism and mitochondrial biogenesis. High-dose naringin restored these proteins to near-normal levels, indicating improved mitochondrial health. Additionally, neurotransmitter levels were favorably influenced: dopamine and serotonin, both severely reduced by d-galactose, were significantly elevated following naringin treatment. The hippocampal histology supported these behavioral outcomes, showing less neuronal damage and structural preservation in treated mice (97).

### 6.2 Suppression of inflammation and endoplasmic reticulum stress

Chronic neuroinflammation and ER stress are major contributors to cognitive deterioration in aging. Dai et al. (98) explored these mechanisms in aged rats and found that naringin administration mitigated cognitive deficits induced by D-galactose. Behavioral assays, including fear conditioning and novel object recognition, confirmed substantial cognitive improvements. On the molecular level, naringin reduced hippocampal levels of pro-inflammatory cytokines such as IL-1β, IL-6, and MCP-1, while increasing antioxidant activity through enhanced GPx. Notably, ER stress markers GRP78, CHOP, and ATF6 were significantly downregulated, indicating that naringin relieved protein misfolding burden. Furthermore, the study identified suppression of the TLR4/NF-κB pathway as a key mechanism, suggesting that naringin exerts neuroprotection through both anti-inflammatory and anti-stress actions (98).

### 6.3 Reduction of protein aggregation in AD and PD models

In *C. elegans* models genetically engineered to express human α-synuclein and Aβ peptides, Zhu et al. (106) observed that naringin delayed disease progression. Treated nematodes exhibited reduced α-synuclein aggregation and improved locomotor behavior, highlighting its therapeutic potential in PD. In a model exposed to 6-OHDA, a toxin that selectively damages dopaminergic neurons, naringin preserved neuronal fluorescence and morphology in BZ555 worms, an effect comparable to that of levodopa. The neuroprotective mechanism was attributed to activation of the DAF-16 pathway, a key transcription factor involved in longevity and stress resistance. The study also showed that naringin failed to extend lifespan in mutants lacking DAF-16, suggesting that its neuroprotective and anti-aging effects are tightly linked to this conserved signaling axis (106).

### 6.4 Retinal neuroprotection and mitochondrial remodeling

While most studies focus on central nervous system benefits, Chen et al. (110) expanded the neuroprotective landscape by investigating retinal degeneration, a sensory component often impaired with aging. In aging mice, long-term oral administration of NAR improved electroretinogram parameters, particularly the a- and b-wave amplitudes, indicating better retinal responsiveness. Morphological analyses revealed that NAR preserved retinal structure, particularly in photoreceptor and inner nuclear layers. Mechanistically, this protection was associated with restored mitochondrial dynamics. The study showed increased levels of MFN2, a mitochondrial fusion protein, and reduced expression of the fission-related DRP1. These changes favored a healthier mitochondrial network. Additionally, NAR reinstated autophagic flux, as evidenced by increased autophagy markers, suggesting that it helps maintain cellular homeostasis in retinal neurons under aging stress (110).

### 6.5 Restoration of neural stem cell function and cognitive ability

A key feature of brain aging is the decline in neural stem cell (NSC) renewal and neurogenesis. Gao et al. (111) identified NAR as a bioactive component within Ribes meyeri anthocyanins and assessed its anti-senescent properties in cultured NSCs and aging mice. *In vitro*, NAR significantly increased cell proliferation, reduced expression of aging markers such as p16INK4a, lengthened telomeres, and promoted neuronal differentiation. These effects translated *in vivo*, where NAR administration improved spatial memory performance in aged mice, as shown in the Morris Water Maze test. The improvement correlated with reduced levels of TNF-α, suggesting that part of its neuroprotective effect stems from dampening systemic inflammation. Enhanced hippocampal neurogenesis further supported its role in restoring cognitive capacity (111).

### 6.6 Cognitive rescue in AD mouse model: SAMP8 study

Further support for NAR’s potential in age-related dementia came from Zhou et al. (99), who studied high-fat diet-fed SAMP8 mice, a widely accepted model of sporadic AD. Over a 12-week period, dietary supplementation with 0.2% NAR significantly improved spatial memory and learning, as shown in both Barnes and Morris maze tasks. Molecular analyses revealed multiple beneficial changes: reduced amyloid-β deposition, decreased tau phosphorylation, lowered oxidative stress markers, and attenuated neuroinflammatory responses. These effects collectively point to a broad-spectrum neuroprotective role for NAR in slowing cognitive decline typical of AD. This study extends the potential application of naringenin beyond general aging to neurodegenerative diseases with complex pathology (99).

### 6.7 Integrative mechanisms of NAR neuroprotection

Across the aforementioned studies, several converging mechanisms emerge. Naringenin consistently enhances mitochondrial health by upregulating biogenesis pathways (AMPK/PGC1α) (97), improving fusion–fission balance (MFN2/DRP1), and restoring autophagy (110). Its ability to decrease oxidative stress and lipid peroxidation while promoting antioxidant enzyme activity has been documented in multiple models. Equally critical is its modulation of neuroinflammation through downregulation of pro-inflammatory cytokines and inhibition of the TLR4/NF-κB axis (98). These anti-inflammatory and antioxidative effects often translate into structural preservation in brain regions such as the hippocampus and retina. In addition, NAR improves neurotransmitter balance, particularly dopamine and serotonin, which supports emotional and cognitive functions. It also increases levels of neurotrophic factors such as NGF and BDNF, enhancing synaptic plasticity and neuronal survival. At the genetic and epigenetic level, it influences transcription factors such as Nrf2 and DAF-16, which are central regulators of cellular defense and longevity pathways (88, 106).

To aid interpretation of preclinical data, it is important to distinguish between molecular and behavioral outcomes. In murine studies, markers such as SOD, CAT, and BDNF reflect mechanistic engagement of antioxidant and neurotrophic pathways, whereas improvements in learning, memory, or motor performance indicate functional relevance. Dose equivalence between animal and human studies should also be considered: using standard body-surface-area conversion, an effective oral dose of 50 mg/kg in mice approximates 4 mg/kg in humans (roughly 250 mg/day for a 60 kg adult). While such extrapolations are only indicative, they help contextualize experimental findings and underscore the need for carefully designed dose-escalation trials in future translational work (112, 113).

Taken together, the evidence from these seven studies underscores the multifaceted neuroprotective effects of NAR in aging and neurodegeneration. Its ability to restore cognitive functions, reduce inflammation and oxidative damage, improve mitochondrial performance, and enhance neurotrophic signaling makes it a strong candidate for further development as a therapeutic or nutraceutical agent. While most data come from preclinical studies, the consistency across species and models—including worms, rodents, and cell cultures—suggests a conserved and robust mechanism of action. These findings advocate for future clinical investigations to determine the translational relevance of naringenin in human cognitive aging and neurodegenerative diseases like AD’s and PD’s.

## 7 Counteracting cardiac aging with NAR: the roles of SIRT1 modulation and mitochondrial channel activation

Cardiovascular aging is characterized by progressive deterioration in cardiac structure and function, largely driven by cellular senescence, oxidative stress, mitochondrial dysfunction, and impaired stress signaling pathways (114). Thus, this part highlights translational implications of naringenin’s mechanistic effects in cardiovascular aging models.

A growing body of research has pointed to the bioflavonoid NAR, derived from citrus fruits, as a promising candidate for counteracting age-related cardiac decline. Emerging studies demonstrate its multifaceted mechanisms of action, particularly in modulating senescence-associated markers and enhancing the expression and activity of SIRT1, a critical regulator of cellular longevity and metabolic integrity. Da Pozzo et al. (30) investigated the anti-aging properties of naringenin in a cellular model of myocardial senescence using H9c2 cardiomyoblasts exposed to hydrogen peroxide to induce premature aging phenotypes. This model mimicked key features of cardiac aging, including β-galactosidase activity, cell cycle arrest, and elevated p16/p21 levels. NAR reduced these senescence markers and oxidative stress, while also limiting DNA damage. It improved mitochondrial metabolic function and calcium buffering in aged cardiomyocytes, suggesting enhanced mitochondrial resilience. Da Pozzo et al. (30) linked these benefits to the activation of mitoBK channels, which may support mitochondrial efficiency and reduce ROS, ultimately protecting against age-related cardiac dysfunction. In a complementary line of research, Testai et al. (91) highlighted NAR’s role as a pharmacological activator of SIRT1, a nicotinamide adenine dinucleotide (NAD+)-dependent deacetylase known to orchestrate cellular stress responses and longevity. *In vitro* assays demonstrated that NAR stimulated SIRT1 activity in a concentration-dependent manner, with efficacy comparable to the established activator resveratrol. Computational modeling further revealed that NAR binds within the same activation sites as resveratrol in the SIRT1 N-terminal domain, forming hydrogen bonds and hydrophobic interactions that enhance enzyme-substrate affinity. The *in vivo* implications of SIRT1 activation by NAR were further validated in aged mice. Chronic oral administration of NAR (100 mg/kg/day for 6 months) to 6-month-old mice led to sustained elevation of myocardial SIRT1 expression by the age of 12 months. This upregulation corresponded with reduced ROS levels in cardiac tissues and significant decreases in systemic inflammatory markers, including TNF-α and IL-6, both of which are known contributors to cardiovascular aging and fibrosis.

Moreover, NAR -treated mice exhibited improved citrate synthase activity and mitochondrial membrane stability, highlighting bioenergetic restoration in aged cardiac tissue. Testai et al. (92) expanded this evidence in 12-month-old rats subjected to ischemia/reperfusion (I/R) injury, a condition exacerbated by aging. In these aged models, the cardioprotective capacity of conventional interventions was markedly diminished. However, pre-treatment with NAR substantially reduced infarct size, an effect abrogated by paxilline, a selective blocker of mitoBK channels. These results not only confirmed the involvement of mitoBK activation in NAR’s protective mechanism but also established the compound’s efficacy in aged myocardium—a context where many cardioprotective drugs lose their effectiveness (92). Electrophysiological assays on isolated mitochondria from aged rats revealed that NAR induced significant depolarization of the mitochondrial membrane potential in a dose-responsive manner, a characteristic signature of mitoBK channel opening. Importantly, the presence of BK-forming α- and β-subunits in both aged cardiac tissue and senescent H9c2 cells confirmed the functional relevance of these channels even in advanced age. This mitochondrial modulation likely supports reduced oxidative burden and preserved energy metabolism in aging hearts (92). An additional dimension to NAR’s protective profile involves its influence on estrogenic signaling, which plays a pivotal role in cardiovascular health, particularly in postmenopausal women. Da Pozzo et al. (30) observed that NAR reversed the H_2_O_2_-induced reduction in estradiol production in cardiomyocytes and restored the expression of estrogen-regulated genes such as ERβ and VDR. These findings suggest that part of the flavonoid’s anti-senescent effects may stem from preservation of endocrine signaling critical to myocardial function. Taken together, these three studies underscore the multifactorial cardioprotective potential of NAR in the context of aging.

Through suppression of oxidative stress, preservation of mitochondrial function, activation of SIRT1, and modulation of hormonal pathways, NAR addresses several converging mechanisms that contribute to cardiac senescence. Its demonstrated efficacy in both *in vitro* and *in vivo* aging models provides a compelling case for further exploration as a nutraceutical agent to promote cardiovascular health during aging. Future studies should aim to clarify the dose-response relationship in human subjects, explore its synergistic potential with other SIRT1 activators or mitochondrial stabilizers, and determine the long-term safety of chronic supplementation. Nonetheless, current evidence positions NAR as a promising natural compound capable of mitigating key aspects of myocardial aging and improving resilience against age-associated cardiovascular dysfunction.

## 8 Pharmacokinetics, metabolic fate, and bioavailability of NAR in aging

Aging modifies physiological systems in ways that significantly influence drug absorption, metabolism, and clearance. To explore how these changes affect naringin, a citrus-derived flavonoid glycoside known for its protective role in age-related diseases, Zeng et al. (31) investigated its pharmacokinetics and metabolic behavior in 20-month-old Sprague-Dawley rats. Using a validated RRLC-QQQ-MS/MS method, they quantified both naringin and its primary aglycone, NAR, across plasma, excreta, and various tissues. Their findings revealed that intact naringin was scarcely detected in systemic circulation, whereas NAR, especially in glucuronide and sulfate forms, predominated. This aligns with previous reports on flavonoid metabolism, where enzymatic hydrolysis in the gut precedes systemic availability. The double-peak concentration profile of NAR observed in plasma suggested delayed intestinal absorption due to slow gastric emptying and possibly enterohepatic recirculation, both of which are often altered in aging. Compared to adult rats, aged animals exhibited a marked delay in the time to peak concentration (Tmax), as well as an extended half-life (t*1*/*2*), indicating prolonged systemic retention, likely due to reduced hepatic and renal clearance capacity as noted by (31). Tissue distribution analysis demonstrated that naringin and its metabolites were most abundant in the gastrointestinal tract, liver, kidneys, lungs, and trachea. The systemic exposure (AUC) and maximum plasma levels (Cmax) of naringenin were approximately double those seen in younger rats, implying greater bioavailability in aged models. Interestingly, gender-related differences were evident: female rats showed significantly higher AUC and longer half-lives than males, possibly due to variations in P450 enzyme activity and renal clearance rates, consistent with findings from Scandlyn et al. (115).

Metabolite profiling via UFLC-Q-TOF-MS/MS revealed extensive phase II biotransformations, including glucuronidation and sulfation. Zeng et al. (31) identified 39 flavonoid conjugates and 46 microbial-derived phenolics in excreta. Naringenin, hippuric acid, and 3-(4’-hydroxyphenyl) propionic acid emerged as dominant metabolites, with most eliminated within 36 h post-dosing. Notably, unmetabolized naringin constituted < 0.01% of the administered dose, confirming near-complete metabolism.

*In vivo* studies indicate that only a small fraction of circulating naringenin reaches brain tissue in its free (aglycone) form, with the majority present as glucuronide or sulfate conjugates. Estimates suggest that conjugated metabolites account for roughly 80–90% of total plasma and brain-associated naringenin, whereas the unbound aglycone typically represents < 10%. This distribution reflects extensive phase II metabolism and limited passive diffusion across the blood–brain barrier (BBB). However, enzymatic deconjugation by β-glucuronidases expressed at the BBB and within glial cells may locally regenerate active naringenin, providing a plausible mechanism for central bioactivity despite low systemic aglycone levels. Recent data also suggest potential involvement of carrier-mediated transport, including organic anion–transporting polypeptides (OATPs), which may facilitate brain entry.

Several formulation strategies have been explored to improve oral absorption and CNS exposure, such as lipid-based nanoparticles, phospholipid complexes, and inclusion complexes with cyclodextrins or solubilizing agents. These systems have been shown to increase naringenin’s apparent solubility, prolong systemic circulation, and enhance brain-to-plasma ratios in rodent models. Such advances underscore the importance of pharmaceutical optimization in translating preclinical neuroprotective findings into clinical settings (31, 116, 117).

In summary, this study underscores that aging enhances systemic exposure to naringin-derived compounds due to altered absorption kinetics and impaired elimination (31). These pharmacokinetic shifts, along with observed sex-specific differences, provide valuable insight for tailoring age-appropriate dosing strategies for NAR -based interventions.

## 9 Systemic and endocrine effects of NAR in aging

Age-related hormonal decline and oxidative imbalance often disrupt pituitary-thyroid regulation. Miler et al. (118) investigated whether dietary flavanones, specifically NAR and hesperetin, could help restore this axis in aged Wistar rats. Their study demonstrated that both compounds influenced gene and protein expression profiles in the hypothalamic–pituitary–thyroid system, with NAR showing stronger effects on several regulatory markers. One of the central findings was the upregulation of SIRT1, a key longevity-related deacetylase. NAR significantly elevated SIRT1 mRNA levels by 91% and its protein expression by 20%, while hesperetin induced increases of 71 and 15%, respectively. This SIRT1 activation in pituitary thyrotrophs suggests a flavanone-induced enhancement in cellular stress response and metabolic regulation (118). Importantly, only NAR lowered the expression of thyroid-stimulating hormone by 20%, indicating a selective influence on the hypothalamic–pituitary–thyroid feedback loop. Both compounds, however, enhanced thyroid peroxidase (TPO) protein expression—essential for thyroid hormone synthesis—by 62% for NAR and 43% for hesperetin. Regarding antioxidant gene regulation in the thyroid, NAR exerted a more complex effect. It suppressed mRNA levels of Tpo, Sod1, Sod2, Cat, and Nrf2 while boosting Gpx expression. This suggests a shift in oxidative defense strategy, potentially relying more on GPx. At the protein level, NAR increased Nrf2 and SOD2 expression by 58 and 50%, respectively, but reduced SOD1 by 48%. In contrast, hesperetin decreased SOD1 and Nrf2 proteins by 63 and 32%, respectively, without increasing any of the studied antioxidant enzymes (118). Collectively, these results suggest that NAR —more than hesperetin—can fine-tune both endocrine signaling and antioxidant defenses in aged organisms. By modulating thyroid function and selectively enhancing antioxidant enzymes, NAR may contribute to restoring redox and hormonal homeostasis disrupted during aging. The findings by Miler et al. (118) provide a mechanistic basis for the beneficial systemic and endocrine roles of citrus flavanones in elderly populations.

## 10 Botanical origins and metabolic enrichment of NAR: insights from aging models

Naringenin, a citrus-derived flavanone with antioxidant and anti-aging properties, is gaining recognition for its role in promoting healthy aging. Its accumulation in certain plant species and its metabolic relevance have recently been elucidated through metabolomic profiling. Two studies—by Zhu et al. (119) and Gao et al. (111)—offer valuable insight into the phytochemical origins and bioactivity of NAR in the context of aging. Zhu et al. (119) conducted a comprehensive metabolomic analysis of *Citri Grandis* Exocarpium (CGE), a fruit peel with known medicinal properties. Samples stored for 1, 3, and 5 years were examined using ultra-high-performance liquid chromatography–quadrupole/time-of-flight mass spectrometry (UHPLC-QTOF-MS/MS). This aging process mimics traditional medicinal preparation and allowed the identification of 1,249 metabolites, including 57 key compounds that varied significantly with storage duration (119). Among these, naringin and its aglycone form NAR were prominently elevated in older samples. Pathway analysis revealed that flavonoid biosynthesis was significantly enriched, with a notable rise in antioxidant-related metabolites such as NAR, butin, and phlorizin over time. These changes suggest that the natural aging of CGE enhances the flavonoid content and antioxidant capacity of the plant material, positioning CGE as a functional source of NAR for nutraceutical or therapeutic use (119). In parallel, Gao et al. (111) explored *Ribes meyeri*, a lesser-known berry species rich in anthocyanins and flavonoids. Through targeted metabolomic profiling, they identified NAR as one of the principal flavonoids contributing to its bioactivity. The researchers evaluated its impact on NSC aging using both *in vitro* and *in vivo* models. Treatment with NAR not only improved NSC proliferation and reduced cellular senescence markers such as *p16^INK4a*, but also enhanced telomere length and DNA synthesis.

Further analysis showed increased expression of Ki67 and MAP2, markers of mitosis and neuronal differentiation, respectively. *In vivo*, aged mice receiving 20 mg/kg of NAR exhibited improved performance in spatial learning and memory tasks. Notably, these cognitive benefits correlated with a significant reduction in systemic TNF-α levels, implicating NAR in the modulation of age-related neuroinflammation (111). Collectively, these studies demonstrate that both *Citri Grandis* and *Ribes meyeri* serve as potent dietary sources of NAR which accumulates over time and contributes to their antioxidant potential. Moreover, the evidence from cellular and animal models confirms the functional relevance of plant-derived NAR in ameliorating hallmarks of aging, including oxidative stress, inflammation, and cognitive decline. These findings support the inclusion of naringenin-rich botanicals in aging intervention strategies.

## 11 Limitations and future directions

Naringenin consistently demonstrates antioxidative, anti-inflammatory, and mitochondrial-supportive properties across diverse experimental models of aging and neurodegeneration. Its effects converge on key signaling cascades such as Nrf2, SIRT1, and AMPK, leading to improved redox homeostasis, proteostasis, and synaptic maintenance. Collectively, these mechanistic observations highlight naringenin as a multitarget modulator of aging biology with strong preclinical support for neuroprotection and systemic homeostasis.

However, several limitations must be acknowledged when interpreting these findings. Most available data derive from animal or *in vitro* studies, and direct human evidence remains lacking. Reported effects are often dose-dependent and may vary according to species, tissue distribution, and experimental design. At higher concentrations, naringenin can exert neutral or even pro-oxidant effects, suggesting a narrow therapeutic range. Interspecies differences in metabolism, intestinal absorption, and conjugation substantially influence bioavailability and blood–brain barrier permeability, making translation to clinical settings uncertain. Moreover, variability in study duration, formulation, and outcome assessment complicates cross-study comparisons. Addressing these issues through standardized methodologies, pharmacokinetic optimization, and cross-species validation will be essential to advance the field.

Noteworthy, the interplay between redox regulation and neurotrophic support represents a key biological link between cellular homeostasis and functional resilience. Naringenin’s ability to modulate BDNF and NGF signaling, primarily through activation of the TrkB–ERK–CREB axis, aligns with broader evidence that antioxidant balance influences neurotrophin expression. Human and experimental findings suggest that oxidative stress reduction can upregulate circulating or brain-derived BDNF levels, promoting neuronal survival and synaptic plasticity (74, 120). These data support the hypothesis that naringenin’s neuroprotective profile extends beyond antioxidant defense to include restoration of neurotrophin-mediated adaptive responses. A similar convergence of redox and mitochondrial mechanisms has been observed for other natural compounds such as N-acetylcysteine and acetyl-L-carnitine, which exhibit complementary antioxidant and neurotrophic effects (121). Such parallels help position naringenin within a broader nutraceutical framework targeting interconnected pathways of oxidative stress, energy metabolism, and neuronal signaling.

This review is narrative in nature and does not apply formal systematic inclusion criteria; thus, the possibility of selection bias cannot be excluded. Although multiple databases were searched, publication bias and heterogeneity among study designs may have influenced the representation of findings. Furthermore, most included data derive from preclinical models, and extrapolation to human physiology must be approached cautiously. Despite these constraints, the synthesis of convergent mechanistic and translational evidence provides a coherent overview of naringenin’s role in modulating aging-related processes. Future systematic analyses and clinical trials are warranted to validate these hypotheses and quantify their clinical relevance. Therefore, these limitations underscore the preliminary nature of current evidence and provide a foundation for the research priorities outlined in the Conclusion section.

## 12 Conclusion

Naringenin emerges as a promising natural flavonoid capable of modulating several interconnected hallmarks of aging, including oxidative stress, mitochondrial dysfunction, and chronic inflammation. Evidence from preclinical studies consistently shows its ability to enhance antioxidant defenses, preserve mitochondrial integrity, and support neurotrophic signaling pathways that maintain cognitive and cardiovascular function. These findings highlight its potential as a multitarget agent for promoting healthy aging and protecting against age-related decline.

However, most available data are derived from animal and cell-based models, and translation to human physiology remains to be validated. Differences in metabolism, bioavailability, and blood–brain barrier permeability may considerably influence its clinical performance. Future research should therefore focus on improving oral and CNS delivery of naringenin, standardizing dosing and formulation approaches, and identifying reliable biomarkers of redox and mitochondrial engagement. Well-designed clinical trials evaluating cognitive, metabolic, and cardiovascular outcomes will be critical to confirm its efficacy and safety in humans. Comparative studies with other flavonoids may further clarify whether the observed benefits are compound-specific or represent a broader class effect.

Taken together, naringenin represents a biologically versatile compound with strong mechanistic support but still preliminary translational evidence. Strengthening the bridge between molecular insight and clinical validation will determine its true potential as a nutraceutical strategy for healthy brain and systemic aging.
